# Evaluation on curative effects of isokinetic muscle strength training for improving muscle function in patients with knee osteoarthritis: A protocol for systematic review and meta-analysis

**DOI:** 10.1097/MD.0000000000027655

**Published:** 2021-11-12

**Authors:** Ji-Qian Wu, Xin-Chong Che

**Affiliations:** Department of Physiotherapy and Hydrotherapy, Taihe Hospital, Shiyan, Hubei, China.

**Keywords:** effective, isokinetic muscle strength training, knee osteoarthritis, muscle function

## Abstract

**Background:**

Knee osteoarthritis (KOA) refers to a chronic deteriorating disease distinguished by degeneration of joint cartilage. Many clinical studies have demonstrated that isokinetic muscle strength training can improve muscle function in patients with KOA. However, although such studies deduce an excellent effect, the results remain controversial. Therefore, the present systematic review seeks to explore the curative effects of isokinetic muscle strength training to establish if it can improve muscle function in patients experiencing KOA.

**Methods:**

This review will entail a systematic review and a comprehensive examination to establish all randomized controlled studies covering curative effects of isokinetic muscle strength training to improve muscle function in patients with KOA. We will obtain data from PubMed, Cochrane Library, EMBASE, China National Knowledge Infrastructure, and WanFang databases from inception to September 2021. In addition, the study will employ the criterion postulated by Cochrane to ascertain a quality evaluation and risk assessment of the studies included for analysis. Also, we will employ relative risk, mean differences, and standardized mean differences with 95% confidence intervals to estimate the effective measures. Also, we will employ Cochran *Q* test and *I*^*2*^ statics to assess heterogeneity among the 2 studies.

**Results:**

Overall, this study anticipates providing accurate results and balanced inferences on curative effects of isokinetic muscle strength training for improving muscle function in patients with KOA.

**Conclusion:**

The study's inferences will offer evidence to decide whether isokinetic muscle strength training is an effective measure for improving muscle function in patients with KOA.

## 1 Introduction

Knee osteoarthritis (KOA) is a chronic joint disease often typified by a deterioration of articular cartilage and secondary bone hyperplasia. In essence, the late disability rate is as high as 53%, which is among the 4 main reasons for the world's disability.^[1]^ The common pathogenesis of KOA is the knee joint, with joint pain as the primary feature. This is followed by the loss of physical functions and muscle weakness, which can initiate degenerative changes in joints and surrounding areas and severely influence the quality of life.^[2–4]^ Treatment of KOA is largely physiotherapy, drug treatment, and surgical treatment. Physical treatment can relieve patient joint pain, improve joint function, and have a slight adverse reaction. Besides, its clinical application is broad and is characterized by lower costs, hence a lighter economic burden for patients. Several studies have affirmed that joint strength training and aerobic exercise can also alleviate joint pain, enhance joint function, and delay the progression of KOA.^[5,6]^

Muscles around knee joints of KOA patients tend to have joint muscle weakness. Also, the pathogenesis of KOA appears to be closely related to muscles, but muscle function is essential in joint sports. It participates in stress absorption, the body feeling, and joint stability. It is also critical to joint protection. In particular, the surrounding muscle function of the knee joint affects the development of KOA.^[7–9]^ Presently, muscle training is utilized as a non-invasive, efficient treatment means, which is increasingly used in muscle functional rehabilitation treatment. It is also widely applied in rehabilitation treatment in improving the muscle strength of the injured joint.

After conducting a preliminary search and analyzing database resources, we established that randomized controlled trials (RCTs) of acupuncture for epididymitis have become more perceptible. Still, most clinical trials tend to be made up of poor quality studies with small sample sizes and insufficient evidence-based examinations due to the limitation of size and number of clinical centers. To this end, we anticipate systematically evaluate the curative effects of isokinetic muscle strength training for improving muscle function in patients with KOA.

## 2 Methods and analysis

We intend to develop the study as per the Preferred Reporting Items for Systematic Reviews and Meta-analysis Protocol (PRISMA-P) guidelines.^[10]^ Also, our study has been registered on OSF (https://osf.io/) under number 10.17605/OSF.IO/Z4M5H.

## 3 Inclusion criteria for study selection

### 3.1 Types of studies

The study intends to collect data on isokinetic muscle strength training for improving muscle function in patients with KOA, regardless of whether such studies are already published or not, and will be based on RCTs method.

### 3.2 Types of participants

We will include only participants diagnosed as KOA per the American College of Rheumatology diagnostic criteria (1986).

### 3.3 Types of interventions

We will also consider the RCTs that include isokinetic muscle strength training as the treatment for inclusion. Also, we will consider all comparisons, including physical therapy, surgery, or other treatments, for inclusion.

### 3.4 Types of outcome measures

The main outcomes include the Lysholm Knee Scoring Scale, peak torque, the average number of adverse effects or events, and serious adverse effects. Likewise, the minor outcomes include total work and visual analogue scale.

## 4 Search methods for the identification of studies

### 4.1 Electronic searches

We intend to perform a systematic and comprehensive examination to find all the randomized controlled studies on curative effects of isokinetic muscle strength training for improving muscle function in patients with KOA. We will obtain information in PubMed, Cochrane Library, EMBASE, China National Knowledge Infrastructure, and WanFang databases from inception to September 2021. The search terms will include “knee osteoarthritis,” osteoarthritis,” “isokinetic muscle strength training,” “isokinetic exercise,” and “isokinetic training.”

### 4.2 Searching other resources

Accordingly, the manual search followed the databases mentioned above to detect all relevant literature.

## 5 Search methods for the identification of studies

### 5.1 Selection of studies

Two independent authors will review all titles and abstracts we identified through the comprehensive search strategy described above. They will compare papers from the same authors, organizations, and countries to minimize data duplication due to multiple reporting. After that, the authors will retrieve full papers for articles established to be eligible based on their titles or abstracts. In addition, all potentially relevant studies selected by at least an author will be retrieved, and the full text will be evaluated further. Only articles that meet the inclusion criterion set by the 2 authors will be assessed. Where there are disagreements between the 2 authors, they will resolve them through discussion.

### 5.2 Data extraction

The following information will be extracted from all included studies: “article information (including author, publication date, and country), demographics information (such as age, sex, and sample size), intervention measures, outcomes, and AEs”. The 2 independent authors will conduct data extraction through a predefined data extraction form to record all studies’ defining features. Where there are disagreements between the 2 authors, they will resolve them through discussion. Finally, we will present extracted data as included study summary table.

### 5.3 Study quality assessment

The criterion put forward by Cochrane will be used for quality and risk assessments of all studies included. This will involve “random sequence generation, allocation concealment, blinding of participants and personnel, blinding of outcome assessment, incomplete outcome data, selective reporting, and other sources of bias”. Each study's methodology will be graded as “high risk,” “low risk,” or “unclear.”

### 5.4 Measures of treatment effect

We will utilize relative risk with 95% confidence intervals as the effect measure for dichotomous data. Accordingly, we will utilize mean differences or standardized mean differences as the effect measure for continuous outcomes.

### 5.5 Assessment of heterogeneity

We intend to utilize Cochran *Q* test and *I*^*2*^ statics to evaluate heterogeneity among the 2 studies. Also, we will use the fixed-effect model to deduce the results if heterogeneity is not statistically significant (*I*^*2*^ < 50% or *P* > .1). Otherwise, we will utilize the random-effect model if heterogeneity (*I*^*2*^ > 50% or *P* < .1).

### 5.6 Sensitivity analysis

We will carry out sensitivity analyses to assess the effect of individual studies on the general influence by excluding 1 study every time from the analysis.

### 5.7 Publication bias

Where the meta-analysis result has more than 10 articles and above, we intend to utilize a funnel plot for testing the risk of publication bias.

## 6 Discussion

KOA is considered one of the widespread diseases in elderly populations. In essence, its induced factors are primarily associated with factors such as age, obesity, damage, and exercise. KOA can result from the exhaustion of various tissues, such as muscles around the joints, leading to changes in the patient's osteoarthritis and muscle mechanics, such as muscle shrinkage function, decreased joints, unstable joints, and balance capacity.^[3]^ From our standpoint, no existing study has compared the efficacy of isokinetic muscle strength training for improving muscle function in patients with KOA. To this end, we suppose that exploring the clinical evidence associated with the effectiveness of isokinetic muscle strength training for improving muscle function in patients with KOA is critical. Thus, the present study will provide evidence for the utilization of isokinetic muscle strength training for improving muscle function in patients with KOA.

## Author contributions

**Conceptualization:** Ji-Qian Wu, Xin-Chong Che.

**Data curation:** Ji-Qian Wu, Xin-Chong Che.

**Formal analysis:** Ji-Qian Wu, Xin-Chong Che.

**Funding acquisition:** Xin-Chong Che.

**Investigation:** Ji-Qian Wu, Xin-Chong Che.

**Methodology:** Xin-Chong Che.

**Project administration:** Ji-Qian Wu, Xin-Chong Che.

**Resources:** Xin-Chong Che.

**Software:** Ji-Qian Wu, Xin-Chong Che.

**Validation:** Ji-Qian Wu, Xin-Chong Che.

**Visualization:** Xin-Chong Che.

**Writing – original draft:** Ji-Qian Wu.

**Writing – review & editing:** Xin-Chong Che.
